# Phosphodiesterase Inhibitors: Could They Be Beneficial for the Treatment of COVID-19?

**DOI:** 10.3390/ijms21155338

**Published:** 2020-07-27

**Authors:** Mauro Giorgi, Silvia Cardarelli, Federica Ragusa, Michele Saliola, Stefano Biagioni, Giancarlo Poiana, Fabio Naro, Mara Massimi

**Affiliations:** 1 Department of Biology and Biotechnology “Charles Darwin”, Sapienza University of Rome, 00185 Rome, Italy; michele.saliola@uniroma1.it (M.S.); stefano.biagioni@uniroma1.it (S.B.); giancarlo.poiana@uniroma1.it (G.P.); 2 Department of Anatomical, Histological, Forensic Medicine and Orthopedic Sciences, Sapienza University, 00185 Rome, Italy; silvia.cardarelli@uniroma1.it (S.C.); fabio.naro@uniroma1.it (F.N.); 3 Department of Life, Health and Environmental Sciences, University of L’Aquila, 67100 L’Aquila, Italy; fragusa@unite.it

**Keywords:** PDE, inflammation, fibrosis, SARS-CoV-2, coronavirus, cytokine storm, cAMP, cGMP, pan-selective inhibitors, pneumonia

## Abstract

In March 2020, the World Health Organization declared the severe acute respiratory syndrome corona virus 2 (SARS-CoV2) infection to be a pandemic disease. SARS-CoV2 was first identified in China and, despite the restrictive measures adopted, the epidemic has spread globally, becoming a pandemic in a very short time. Though there is growing knowledge of the SARS-CoV2 infection and its clinical manifestations, an effective cure to limit its acute symptoms and its severe complications has not yet been found. Given the worldwide health and economic emergency issues accompanying this pandemic, there is an absolute urgency to identify effective treatments and reduce the post infection outcomes. In this context, phosphodiesterases (PDEs), evolutionarily conserved cyclic nucleotide (cAMP/cGMP) hydrolyzing enzymes, could emerge as new potential targets. Given their extended distribution and modulating role in nearly all organs and cellular environments, a large number of drugs (PDE inhibitors) have been developed to control the specific functions of each PDE family. These PDE inhibitors have already been used in the treatment of pathologies that show clinical signs and symptoms completely or partially overlapping with post-COVID-19 conditions (e.g., thrombosis, inflammation, fibrosis), while new PDE-selective or pan-selective inhibitors are currently under study. This review discusses the state of the art of the different pathologies currently treated with phosphodiesterase inhibitors, highlighting the numerous similarities with the disorders linked to SARS-CoV2 infection, to support the hypothesis that PDE inhibitors, alone or in combination with other drugs, could be beneficial for the treatment of COVID-19.

## 1. Introduction

Coronavirus disease 19 (COVID-19) is caused by infection with the severe acute respiratory syndrome corona virus 2 (SARS-CoV2), a virus belonging to the vast family of coronaviruses, responsible for illnesses ranging from the common winter cold to more serious diseases, such as the Middle East respiratory syndrome (MERS-CoV) and severe acute respiratory syndrome (SARS) [1].

The COVID-19 virus infection begins when SARS-CoV-2 virions come into contact with epithelial cells of the respiratory mucous membranes that, together with the goblet cells, form the first and effective protective barrier. Entry of the virus into the host cell is mediated by the surface spike protein that binds to the angiotensin-converting enzyme 2 (ACE2) receptors. The spike protein is one of four structural proteins of the SARS-CoV-2 capsid; the others are the envelope, the membrane and the nucleocapsid proteins. The mechanisms of the entry of the virus into the cell have already been reported in great details [2], while the immediate consequence of the infection is the activation of the innate immunity reaction, decisive for the outcome of the infection, with negligible or very severe effects [3]. Severely affected patients often go through the acute respiratory distress syndrome (ARDS), characterized by acute inflammation and extensive lung damage. Although inflammation can help fight infection, when out of control, it can generate accumulation of cytokines. An excessive accumulation of cytokines can cause a storm, with severe damage of tissues and multiple organ failure [4]. Therefore, inhibition of excessive inflammation is a fundamental action to avoid the spreading of damage in tissues in SARS, and probably also in COVID-19.

It follows that the high levels of inflammatory cytokines, such as inteleukin-6 (IL-6), tumor necrosis factor (TNF), interleukin-1 (IL-1), chemokines and the prognostic significance of IL-6 levels provide a solid rationale for adopting strategies that include treatment with anti-IL-6 monoclonal antibodies or anti-IL-16R (e.g., Tocilizumab), anti-IL-1 (e.g., Canakinumab), recombinant IL-1 receptor antagonist (anakinra) or inhibitors of cytokine signaling pathways, such as janus kinases 1 and 2 (JAK1,2) (e.g., baricitinib). In this context, multitarget strategies appear to be the most promising actions to control inflammation [5,6].

To date, the only agent for which more reliable data are available is Tocilizumab, a humanized monoclonal antibody against the IL-6 receptors. Currently, in China and other parts of the world, studies are underway to approve its use in COVID-19. This drug is already in use for the control of inflammation in rheumatoid arthritis, and in the cytokine release syndrome in antigen receptor T (CAR-T) cell therapy [7]. Therefore, at the moment, most adopted strategies aim to combat inflammation to avoid the dramatic effects of post-SARS-CoV-2 infection. Corticosteroids and nonsteroidal anti-inflammatory drugs (NSAIDs) have given encouraging results in the short-term; however, when used for a longer time, possible multiple adverse events may prevent their use on a larger scale for all patients [8,9]. Therefore, finding new drugs for the control of inflammation represents a very coveted goal in scientific research and, in particular, in the control of COVID-related pathologies [10,11].

Scientists around the world have also observed a close correlation between SARS-CoV-2 infection and blood clotting disorders, in patients not considered at risk and without a stroke history. In some cases, even young adult patients suffered from large-vessel stroke in association with SARS-CoV-2, while these thrombotic complications appear to drop significantly in the youngest patients [12,13,14].

The average age of hospitalized patients with severe COVID-19 is higher than the average age of patients with MERS or SARS; moreover, approximately 40% of COVID-19 patients develop acute respiratory distress syndrome (ARDS) and 20% of total ARDS are severe cases [15].

Since interstitial pulmonary fibrosis is believed to be a direct consequence of ARDS, and to be also responsible for an irreversible process that leads to the loss of lung functions, old age could be an additional risk factor for the development of pulmonary fibrosis, as a consequence of inflammatory pulmonary processes, including those associated with autoimmune disease [16]. Similar repercussions had already been observed in people affected by SARS, who showed clearly visible anomalies associated with reduced lung volume and breathing capacity six months after infection [17].

In light of these observations, thwarting the development of pulmonary fibrosis after COVID-19 recovery could be of great interest for public health. The removal of the causes of lung damage does not in itself prevent the development of progressive and irreversible interstitial lung fibrosis. In fact, even following less severe infections, characterized by mild and apparently non-progressive fibrosis, the degree of morbidity and mortality could be high in elderly COVID-19 patients, especially with pre-existing lung conditions. Although at the moment the long-term lung repercussions of COVID-19 remain speculative and require adequate prospective studies, the huge number of individuals affected by COVID-19 suggests that this aspect should not be underestimated, since it could have serious health consequences [18,19].

## 2. Cyclic Nucleotide Pathway in Inflammation and Fibrosis

In higher eukaryotes the second messengers cyclic guanosine monophosphate (cGMP) and cyclic adenosine monophosphate (cAMP) are signal molecules of major transduction pathways fundamental in the regulation of multiple cellular functions. Phosphodiesterases (PDEs), a superfamily of cAMP/cGMP hydrolyzing enzymes, have a major role in the fine regulation of cyclic nucleotide concentration within cells [20].

To date, eleven PDE isozyme families encoded by 24 distinct genes have been identified in mammals displaying distinct biochemical properties, including substrate specificity and tissue specific expression. The PDE4, 7 and 8 isozyme families can only hydrolyze cAMP; PDE5, 6 and 9 are specific for cGMP, while all the other members have dual substrate specificities [21].

Subfamilies of cAMP- and cGMP-selective PDEs, differentially distributed in cells and tissues are the subject of intense pharmacological research in the field of inflammation, cognition, lipogenesis and fibrosis, as well as of cell differentiation, apoptosis, proliferation and cancer [22,23,24,25].

Among the different PDEs, the cAMP-specific PDE4 is highly expressed in cardiovascular tissues, smooth muscles, brain, keratinocytes and immune system cells, including T cells, monocytes, macrophages, neutrophils, dendritic cells and eosinophils [26]. Reported data show that inhibition of PDE4 can effectively raise the intracellular level of cAMP, and help to modulate inflammatory and immune system responses [27]. At present, PDE4 represents an effective therapeutic strategy in many inflammatory conditions, including asthma, chronic obstructive pulmonary disease (COPD), idiopathic pulmonary fibrosis, psoriasis, atopic dermatitis (AD), inflammatory bowel disease (IBD), rheumatic arthritis (RA), lupus and inflammation of the nervous system [28,29]. PDE4 inhibitors, such as roflumilast, apremilast and crisaborole have also been approved in succession for the treatment of the inflammation of the respiratory tract, as well as for a variety of skin diseases [30].

Several PDE4 inhibitors have been developed for the treatment of inflammation with promising therapeutic efficacy. Many of these newly synthesized PDE4 inhibitors, already in advanced preclinical studies, show a large anti-inflammatory and broad-spectrum outcome. Thanks to the wide tissue distribution of this PDE, its inhibition can reduce the inflammatory response induced by macrophages, dendritic cells (DC), T helper Th1, Th2 and Th17 cells, increasing the production of macrophage anti-inflammatory cytokines, which can interfere with the phenotype and functions of B lymphocytes [30].

In various cell models, it has also been shown that cAMP has marked anti-fibrotic effects [31], and is directly involved in the regulation of cellular functions of lung and bronchial cells, as well as of the functions of cells implicated in inflammatory processes. Elevated cAMP levels also promote relaxation of lung smooth muscle cells (ASMC) [32] and reduce the proliferation and migration of ASMC cells and/or lung fibroblasts, as well as their ability to synthesize extracellular matrix proteins. In addition, high levels of cAMP strongly reduce the transition of fibroblasts to myofibroblasts [33]. All these observations lead the way to increased interest in PDE inhibitors for the control of respiratory diseases [34,35],

However, the most effective inhibitors could be those with broad range that do not inhibit specific isoforms, but are inhibitors of various PDEs, i.e., pan inhibitors. Indeed, the first non-selective PDE inhibitor used in the treatment of asthma was theophylline (1,3-68 dimethylpurin-2,6-dione), a compound belonging to the methylxanthine group, natural molecules very similar to cyclic nucleotides that can be considered the archetypes of pan-selective inhibitors [36]. Although a few side effects have limited its therapeutic application on a large scale, recently, theophylline has been proposed for the treatment of pediatric respiratory tract diseases [37].

Since it has been shown that various PDEs, i.e., PDE1, PDE3, PDE4, PDE5 and PDE7, are implicated in the pathogenesis of asthma or in COPD [27,34], research has been directed to develop inhibitors that could affect the various isoforms, while maintaining high selectivity for PDEs. The purpose of this approach is not only to obtain a more precise inhibition of the enzymes, but also to reduce the severe side effects that characterize less selective inhibitors. As previously mentioned, several inhibitors have undergone clinical trials, and some of them, including the PDE4 inhibitor roflumilast, are currently in clinical use [38]. Another example is the inhaled PDE4 inhibitor, CHF 6001, currently undergoing phase II clinical trials, which has effective anti-inflammatory properties in COPD [39].

At present, the efficacy of inhibitors in many pathologies can hardly be further improved by focusing on inhibitors of individual isoforms, even if they are always more and more selective; the attention of researchers has therefore shifted towards dual or even pan-selective PDE inhibitors. The compartmentalized intracellular localization of individual PDEs, their cell-specific expression, as well as the fact that the expression of individual PDEs may change in response to substances and irritants to which patients may be exposed, including those that cause exacerbations in asthma and COPD, such as smoking tobacco and potentially also COVID-19, are in favor of molecules able to affect multiple simultaneous targets and, at the same time, multiple specific PDE subtypes [40].

Very interesting studies have revealed that compared to selective PDE4 inhibitors (roflumilast or cilomilast), pan-PDE inhibitors give a better inhibition of transforming growth factor type β1 (TGF-β1)-induced ASMC remodeling [41]. The same authors report that the recently synthesized 7,8-disubstituted purine-2,6-dione derivatives, in addition to being pan-selective PDE inhibitors, can interact with “transient receptor potential ankyrin 1” (TRPA1) ion channels [42]. These receptors are non-selective calcium permeable channels expressed in both immune and lung structural cells, in epithelial cells, in smooth muscle cells and in fibroblasts, as well as in sensory neurons. They can be activated by many different irritants, such as allyl isothiocyanate, allicin or acrolein, functioning in this case as toxin sensors, but also by viral infections and pro-inflammatory mediators, including histamine, prostaglandins or bradykinin [43,44]. Their activation is responsible for allergic reactions, hyperresponsiveness of the airways, bronchoconstriction, neurogenic inflammation and cough. Therefore, TRPA1 antagonists may also represent a therapeutic alternative for lung diseases [45].

Wójcik-Pszczoła and coauthors [33] used three different 7,8-disubstituted purine-2,6-dione derivatives (compound “832”—a pan-selective PDE inhibitor, compound “869”—a TRPA1 modulator and compound “145”—a pan-selective PDE inhibitor and a TRPA1 modulator) and evaluated their ability to inhibit the pro-fibrotic responses of lung fibroblast cell lines and MRC-5 104 cells once activated by TGF-β1 or FBS. In parallel, they studied their proliferation, migration, contraction, expression of profibrotic genes and phenotypic transition into myofibroblasts. The data show that compound “145” exerted the most remarkable effect in limiting the transition from fibroblasts to myofibroblasts (FMT), as well as proliferation, migration and contraction. The data also show that the effects of this compound depend essentially on its strong PDE inhibitory properties and not on its effects on the modulation of TRPA1. The strong anti-remodeling effects of “145” requires activation of the cAMP/protein kinase A/CREB pathway, leading to the inhibition of TGF-β1 and Smad-dependent signaling. These data suggest that the TGF-β pathway is an important target for PDE inhibitors, which leads to inhibitory effects on cellular responses involved in airway remodeling. These potent pan-selective PDE inhibitors represent promising anti-remodeling drug candidates for further research, including research on anti-COVID-19 drugs [33]. Among these pan-selective compounds, sulindac, originally used as anti-inflammatory drug, was shown to be a PDE5 and PDE10 inhibitor [46]. This molecule used for blocking cell proliferation might be efficiently employed for the control of COVID-19 related inflammation [47,48].

## 3. Cyclic Nucleotide Pathway in Vascular Resistance, Thrombosis and Stroke

Pulmonary vascular dilatation and remodeling are on the other hand controlled at least in part by phosphodiesterase 5 (PDE5), specific for the cyclic nucleotide cGMP. PDE5 has a relatively high expression in airways and vascular smooth muscle, and is believed to produce its effects through the modulation of cGMP-PKG signaling. PDE5 inhibitors, such as sildenafil (marketed as Revatio by Pfizer), tadalafil (marketed as Adcirca by Eli Lilly) and vardenafil (marketed as Levitra by Bayer) are currently approved for the treatment of pulmonary arterial hypertension [49]. In particular, sildenafil, initially used for the treatment of erectile dysfunction, such as in Viagra [50], has more recently been shown to be effective, at different dosages, in improving oxygen intake, heart failure and pulmonary hypertension, with generally mild side effects. Sildenafil has also been shown to have cardioprotective effects in animal and human models [51,52]. The main molecular mechanisms include the enhancement of nitric oxide- cGMP pathway [53], the phosphorylation of ERK [54], as well as the regulation of protein kinase C (PKC) [55], Ras homolog family member A/ Rho-associated protein kinase (RhoA/ROCK) pathways [56] and adrenergic signaling [51]. A recent study has shown that, although sildenafil is not effective in reducing the filling pressure in patients with myocardial infarction, it does, however, manifest beneficial hemodynamic effects on the secondary endpoints in these patients, improving cardiac output (CO), diastolic blood pressure and resistance of vascular vessels [57].

The cardioprotective effects of sildenafil are well-documented in local myocardial ischemic models with left coronary artery ligation [58]. Coronary artery ligation always induces local ischemic injury of the myocardium, differentiating from cardiac arrest resuscitation (CAR) that can instead induce global hypoperfusion, which can cause damage to other organs, including the brain and kidneys [59,60].

Dual-substrate specific PDE inhibitors, in particular PDE3 inhibitors, are also currently receiving a lot of interest [35,61], especially after the discovery that increase of cGMP can reduce oxidative stress in COPD [62]. In addition, cigarette smoking, and thus nicotine, has also been shown to be associated with a decrease in guanyl cyclase levels, the enzyme responsible for the synthesis of cGMP [63] and to induce the expression of PDE3 and PDE4 in the lung [64]. These observations, besides indicating that PDEs are involved in COPD, also provide further confirmation of the validity of the use of PDE inhibitors in the treatment of other lung diseases.

Finally, the use of substance V, a novel and only recently described PDE3 inhibitor, has also proven effective in reducing the volumes of heart attacks and in improving neurological outcomes after experimental cerebral ischemia, with modalities of action that appear to be independent from platelet function. Thus, the pharmaceutical inactivation of PDE3 could also represent a different therapeutic approach to combat ischemic stroke, by reducing blood-brain barrier damage, brain tissue inflammation and local cell death [65].

As we have already said, one of the aspects of COVID-19 is the induction of blood clotting. Additionally, in this case, phosphodiesterase inhibitors could represent good allies, since appreciable action have already been demonstrated as platelet aggregation inhibitors. The platelet aggregation inhibitors currently in use act by interfering with specific phases of the platelet activation process. Platelet inhibition can occur by blocking membrane receptors such as the receptor for fibrinogen and von Willebrand factor (GpIIb/IIIa glycoprotein), the adenosine diphosphate receptor (ADP) P2Y12 or the thrombin platelet receptor (proteinase activated receptor, PAR-1). Alternatively, inhibition of platelet aggregation can be achieved by interfering with intracellular signaling, via modulation of the levels of specific molecules or cytoplasmic messengers, such as arachidonic acid and, in particular, cAMP. In this context, the role of phosphodiesterases in the regulation of platelet function, which express different PDE families, including PDE2, PDE3 and PDE5, is well known. PDE inhibitors, such as cilostazol and dipyridamole, lower platelet activity by increasing cAMP and/or cGMP levels [20,27].

Specifically, cilostazol is a powerful anti-platelet agent, registered in Japan in 1988 and in the United States in 1999, and is currently in use in many other countries for the treatment of intermittent claudication, and in patients with peripheral vascular disease [66]. This pathology can severely limit a person’s ability to walk, and is generally also associated with arterial occlusive disease [67]. Cilostazol has also been tested in randomized controlled trials for secondary stroke prevention [68], cardiovascular disease [69,70] and post-stent stenosis [71,72]. The mechanism by which cilostazol improves intermittent claudication is not fully understood, but probably involves several processes, among which the specific and selective inhibition of PDE3 in platelets seems to be important (IC_50_ = 0.2 μM) [71]. Cilostazol also inhibits the uptake of adenosine enhancing the interstitial concentration of this nucleoside, with a consequent increase in intracellular cAMP [73].

Dipyridamole, initially used as a coronary vasodilator, also exhibits several important anti-platelet effects [74,75]. One of these includes inhibition of PDE5, which, in turn, leads to an increase in cGMP levels [76,77]. In addition, dipyridamole blocks the red blood cells-mediated absorption of the vasodilator adenosine and stimulates the production of prostacycline [77,78].

## 4. Phosphodiesterase Inhibitors in COVID-19

**Figure 1:**
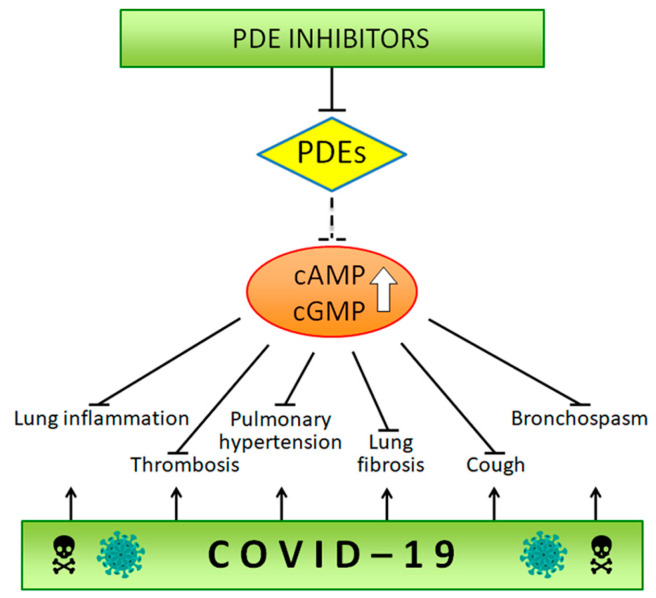
The scheme highlights the different pathologies currently treated with phosphodiesterase inhibitors that are in common with disorders linked to severe acute respiratory syndrome corona virus 2 (SARS-CoV2) infection.

In the last few months, as expected, a few authors have proposed the use of PDE inhibitors for the treatment of COVID-19, based on the clinical features observed in this disease, as well as on their analogy to other already known pathologies, for which the use of inhibitors has already been approved (Figure 1).

Solaimanzadeh and coauthors [79] propose the use of PDE5 inhibitors, showing the analogy of COVID-19 to HAPE (high altitude pulmonary edema), a respiratory disease in which these molecules have already been clinically tested and used. Given the medical emergency due to the growing contagion and the thousands of lives at stake, drugs like acetazolamide, nifedipine and phosphodiesterase 5 inhibitors represent an opportunity not to be underestimated for SARS-CoV-2 infection [79].

PDE5 inhibitors, and sildenafil in particular, have also been proposed, by Isidori and collaborators [80], as modulators of the NO-cGMP-PDE5 axis. This axis has also been considered as a target for phase three trial studies, in consideration of the fact that PDE5 is predominantly expressed in the lungs, the organ most affected by COVID-19. Additionally, as described in the literature and reported in this review, its inhibition may counteract Ang-II-mediated downregulation of the angiotensin II receptor type 1 (AT-1) receptor, reducing pro-inflammatory cytokines, infiltration and alveolar hemorrhage-necrosis. Different formulations of PDE5 inhibitors have shown a good safety profile and reduced mortality in patients with type 2 diabetes mellitus and high cardiovascular risk [52,81]. In addition, sildenafil and tadalafil also inhibit the transition of endothelial and smooth muscle cells to mesenchymal cells in the pulmonary artery, preventing clotting and thrombotic complications [80].

As known, the most serious manifestation of COVID-19 is characterized by a hyperinflammatory state, due to an uncontrolled and massive release of pro-inflammatory cytokines, called a “cytokines storm “. As reported in the literature, and also highlighted in this review, an effective inhibitory action on proinflammatory cytokines is also performed by cAMP, through modulation of protein kinase A (PKA) and nuclear factor κB (NF-κΒ) pathways. In a recent commentary, Dalamaga and co-authors [82] hypothesize that the selective inhibition of cAMP-specific PDE4, a widely distributed PDE whose inhibition leads to appreciable anti-inflammatory, effects in a wide range of cells, may in fact represent a very valid treatment since it can block the initial phase of the inflammatory response, by inhibiting pro-inflammatory cytokines, such as TNF-α, and inducing anti-inflammatory cytokines, such as IL-10, thus, preventing the storm of cytokines responsible for the well-described and serious multiorgan dysfunctions [82]. Of interest, the PDE4 inhibitor apremilast has also been shown to be beneficial in enhancing lipolysis, increasing insulin sensitivity and reducing the accumulation of adipose tissue in the liver, especially in patients with high glycated hemoglobin. This is of particular interest considering that obesity and type 2 diabetes mellitus have been reported as risk factors for COVID-19 severity [82,83].

Bridgewood and coauthors [84] also support the use of PDE4 inhibitors, emphasizing their excellent safety profile and the fact that these agents do not seem to be associated with exacerbations of viral infections, an aspect not to be underestimated considering the advanced age of patients [84].

## 5. Conclusions

There are numerous research studies that support the use of PDE inhibitors, in particular the use of inhibitors of isoforms 4 and 5, in pathologies associated with COVID-19, mostly thanks to their well-documented anti-fibrotic and anti-inflammatory effects. Most of the inhibitors are currently in clinical development, and some of them are already in clinical use for other pathologies and are generally very well tolerated. They could therefore represent effective and at the same time rapid and economical responses to curb COVID-19 and block, or at least slow down, its progression towards the most severe stages. All this is relevant given the medical emergency and the enormous economic impact of the pandemic worldwide.

PDE inhibitors are also of particular interest, and they can effectively guarantee the inhibition in unison of the hydrolyzing activities of many cellular PDEs, thus ensuring high levels of cAMP accumulation in the cell. They also show efficacy toward PDEs considered relevant for the pathogenesis of respiratory diseases.

In conclusion, due to the pleiotropic mechanisms of action of specific PDE inhibitors, well supported by the rich scientific literature, and considering their wide availability in the pharmaceutical market, it is our opinion that exploring the therapeutic potential of PAN-selective inhibitors or cocktails of PDE-specific inhibitors, also in support of other therapeutic regimens, represents a priority in coronavirus research.
